# Contribution of Red Blood Cells and Platelets to Blood Clot Computed Tomography Imaging and Compressive Mechanical Characteristics

**DOI:** 10.1007/s10439-024-03515-y

**Published:** 2024-04-25

**Authors:** Rachel M. E. Cahalane, Janneke M. H. Cruts, Heleen M. M. van Beusekom, Moniek P. M. de Maat, Marcel Dijkshoorn, Aad van der Lugt, Frank J. H. Gijsen

**Affiliations:** 1 Department of Biomedical Engineering, Thoraxcenter, Erasmus MC, Rotterdam, The Netherlands; 2 Experimental Cardiology, Erasmus MC, Rotterdam, The Netherlands; 3 Department of Hematology, Erasmus MC, University Medical Center Rotterdam, Rotterdam, The Netherlands; 4 Department of Radiology and Nuclear Medicine, Erasmus MC, University Medical Center Rotterdam, Rotterdam, The Netherlands; 5 Department of Biomechanical Engineering, Delft University of Technology, Delft, The Netherlands

**Keywords:** Thrombus, Blood clot analogue, Composition, Mechanics, Imaging

## Abstract

Thrombus computed tomography (CT) imaging characteristics may correspond with thrombus mechanical properties and thus predict thrombectomy success. The impact of red blood cell (RBC) content on these properties (imaging and mechanics) has been widely studied. However, the additional effect of platelets has not been considered. The objective of the current study was to examine the individual and combined effects of blood clot RBC and platelet content on resultant CT imaging and mechanical characteristics. Human blood clot analogues were prepared from a combination of preselected RBC volumes and platelet concentrations to decouple their contributions. The resulting clot RBC content (%) and platelet content (%) were determined using Martius Scarlet Blue and CD42b staining, respectively. Non-contrast and contrast-enhanced CT (NCCT and CECT) scans were performed to measure the clot densities. CECT density increase was taken as a proxy for clinical perviousness. Unconfined compressive mechanics were analysed by performing 10 cycles of 80% strain. RBC content is the major determinant of clot NCCT density. However, additional consideration of the platelet content improves the association. CECT density increase is influenced by clot platelet and not RBC content. Platelet content is the dominant component driving clot stiffness, especially at high strains. Both RBC and platelet content contribute to the clot’s viscoelastic and plastic compressive properties. The current in vitro results suggest that CT density is reflective of RBC content and subsequent clot viscoelasticity and plasticity, and that perviousness reflects the clot’s platelet content and subsequent stiffness. However, these indications should be confirmed in a clinical stroke cohort.

**Supplementary Information:**

The online version contains supplementary material available at 10.1007/s10439-024-03515-y.

## Introduction

Thrombotic emboli are one of the main causes of large vessel occlusion acute ischaemic stroke (AIS), of which endovascular thrombectomy (EVT) is the current standard of care [22]. AIS thrombi are diverse in their composition [3, 24] which is known to affect the thrombus mechanics [3, 4]. The mechanical interaction between the thrombus and the EVT retrieval device governs the subsequent EVT success [16]. Platelets and red blood cells (RBCs) are two of the primary components of blood. Healthy adult concentrations of platelets in blood span the range of 150–400 × 10^3^ cells/µl and RBCs account for 40% of the total blood volume. In haemostasis, platelets mediate clot contraction by pulling on fibrin fibres [14], expelling serum from the clot and reducing the clot size. During this platelet-driven clot contraction, RBCs are redistributed and undergo compressive deformation to form polyhedrocytes, reducing clot permeability to prevent blood loss [32]. Red blood cell (RBC)-rich thrombi are less stiff [15] and are associated with higher reperfusion rates [18, 34]. Beyond RBCs, a high thrombus platelet content (> 70%) [3] and platelet-mediated clot analogue contraction [15] also contribute to stiffer thrombi. However, the interdependence of RBCs and platelet content on thrombus mechanics has not been considered.

Most studies to date that examine cellular contributions to clot analogue mechanics utilise two methods of blood clot analogue formation. First, volumes of RBCs and platelet-rich plasma (PRP) can be combined in differing RBC volumetric percentages [5]. Typically, the PRP platelet concentration is not measured or controlled across the RBC volumetric percentages, inadvertently resulting in inconsistent platelet concentrations (as RBC volume increases, platelet concentration decreases). Second, volumetric fractions of RBCs and either platelet-rich plasma (PRP) or platelet-poor plasma can be combined producing dichotomized ‘contracted’ and ‘non-contracted’ clot phenotypes [15]. Overall, contracted clots are stiffer than their non-contracted counterparts for all RBC volume fractions [15]. Since stroke patients do not have thrombocytopenia, platelet-poor plasma clots may not be physiologically relevant in the context of AIS.

Thrombus imaging characteristics at emergency neuroimaging, performed for the diagnosis of ischaemic stroke [35], may be leveraged to predict thrombus mechanics and EVT effectiveness. CT is the most promising neuroimaging modality for pre-treatment thrombus characterisation as it is already performed at most stroke centres. RBC content has already been shown to be positively associated with non-contrast computed tomography (NCCT) density [20, 29]. Platelet content (not considering the RBC content) is weakly negatively associated with NCCT density [9, 20]. Reported associations of thrombus fibrin/platelets conglomerations or RBC content with contrast agent perviousness (CT Angiography density minus NCCT density) have been conflicting [4]. Similar to the mechanical stiffness, these studies did not consider the interdependence of RBCs and platelets in the thrombi. Further investigation on the association of thrombus composition and CT characteristics, particularly perviousness, has been recommended [30].

The individual impact of RBCs on imaging and mechanical properties of thrombi have been explored before. However, in thrombi, RBCs exist in combination with platelets. How the added presence of platelets modulates RBC associations with imaging and mechanical properties is unknown. The current study hypothesises that the presence of platelets modulates the clot structure through an increased contraction and that this will affect the mechanical behaviour of the clots and be reflected in the CT imaging characteristics. Considering these two primary cellular components of blood clots individually versus combined will likely shed light on conflicting findings in the literature. Therefore, the aim of the current study is two-fold. First, to produce clot analogues with RBC and platelet contents representative of thrombi retrieved from AIS. Second, to utilize these clots to examine the interdependent effect of a range of RBC and platelet content on both the CT imaging and compressive mechanical characteristics. This builds upon previous efforts to mimic the histopathological content of thrombi retrieved from AIS patients, that did not conduct imaging or mechanical characterization of their clots [8].

## Materials and methods

### Sample preparation

**Fig. 1:**
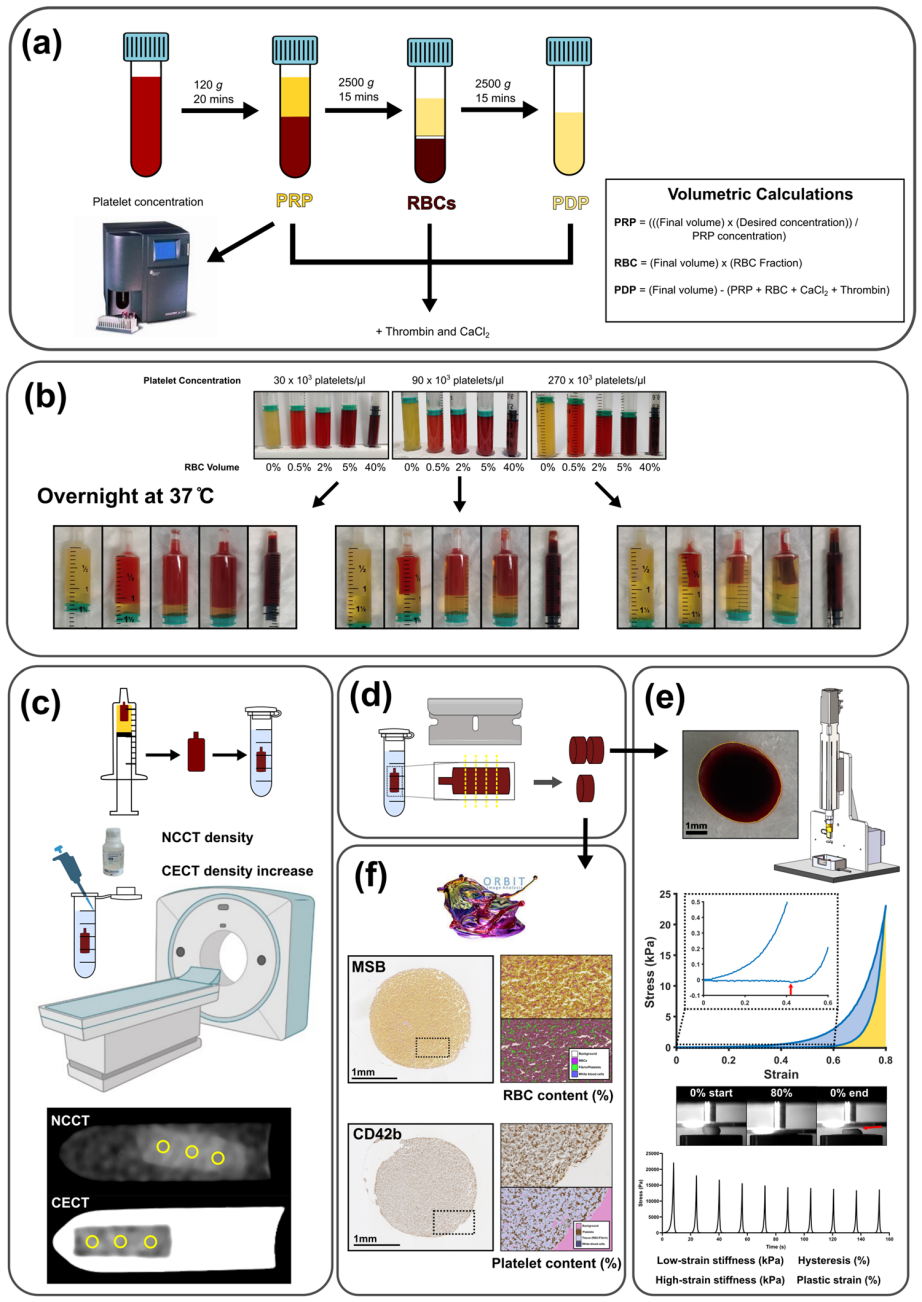
Experimental overview. **a** Citrated whole blood processing and volumetric calculations for platelet-rich plasma (PRP), red blood cells (RBCs), and platelet-depleted plasma (PDP). **b** 15 reconstructed blood sample incubation overnight and contracted clots in syringes the following day. Differences in levels of contraction are observed across the 3 platelet concentrations (30, 90, 270 × 10^3^ platelets/µl) for the 5 RBC volumes (0%, 0.5%, 2%, 5%, 40%). **c** Clot computed tomography (CT) imaging. Non-contrast and contrast-enhanced CT (NCCT and CECT) were performed, and representative regions of interest were selected from the images for density measurements. NCCT density CECT density increase measurements were calculated. **d** Clots were cut to produce three sections from the centre of the clot for mechanical testing (*n* = 2) and histology and immunohistochemistry (*n* = 1). **e** Compression testing. Cross-sectional area measurements were performed in ImageJ. Ten cycles of 80% strain at a rate of 10%/s were performed in a custom compression setup. Representative first loading and unloading cycle stress-strain curve. The percent hysteresis loss was defined as the dissipated energy observed after the first loading–unloading cycle (blue area) expressed as a percentage of the supplied energy through the loading cycle (blue plus yellow areas). The amount of plastic compressive strain (%) was also identified as the point where the amount of stress returned to baseline (indicated by red arrows). Representative 10 stress-time cycles are shown. **f** Histology and immunohistochemistry. Martius Scarlet Blue (MSB) is used to quantify RBC content. CD42b is used to quantify platelet content. Quantification is performed on Orbit Image Analysis Software

Blood was drawn by venepuncture from healthy human volunteers into 0.109 M sodium citrate tubes (BD Vacutainer®). Approval for the use of human material was granted by the Dutch Medical Ethical Testing Committee (METC, NL76853.078.22). The blood was centrifuged, first at 120 g for 20 min at room temperature to separate the platelet-rich plasma (PRP). The PRP platelet concentration was measured using a Coulter Counter (COULTER® AC•T diff™ Analyzer, Beckman Coulter, CA). The remaining material was spun at 2500 g for 15 min at room temperature to separate platelet-poor plasma, buffy coat and red blood cells (RBCs). The buffy coat layer was discarded. Platelet-poor plasma was spun for a second time at 2500 g for 15 min to separate platelet-depleted plasma (PDP) [17] (Fig. 1a). We performed some preliminary work to determine what RBC volumes and platelet concentrations would produce clots with a physiologically relevant range of RBC and platelet content (Figs. S1-S2). Based on these preliminary results, we selected 3 different platelet concentrations (30, 90, and 270 × 10^3^ platelets/μl) and 5 different volumetric RBC percentages (0%, 0.5%, 2%, 5%, and 40%). Coagulation was induced by adding CaCl_2_ (C5670, Sigma-Aldrich) and thrombin (T7009, Sigma-Aldrich) to final concentrations of 17 mM and 1 U/ml, respectively. The calculations for determining PRP, RBC and PDP volumes are presented in Figure 1a and are determined by the PRP platelet concentration as measured above. Additionally, representative volumes are presented in Table S1. The reconstructed blood was immediately transferred into syringes and placed vertically in a 37 °C water bath to fully contract overnight. The 0%, 0.5%, 2% and 5% RBC volume clots were transferred into 3 ml syringes, and the 40% RBC volume clots were transferred into 1 ml syringes to account for the differences in the expected degree of contraction (Fig. 1b).

### Computed tomography imaging

The clots were removed from the syringes and placed into Eppendorf tubes containing Dulbecco’s Modified Eagle Media with no phenol red (Gibco^TM^). Scanning was performed on a dual source CT scanner (384 (2 × 192) slices, Somatom Force, Siemens). The clots were scanned with 120 kVp, a rotation time of 1.0 s and 0.55 pitch. Image reconstructions were made with a field of view of 60 mm, voxel size of 0.12 mm, slice thickness of 0.5 mm and Hv40 convolution kernel. A non-contrast CT (NCCT) scan was performed first to assess the clot CT density. As a proxy for clinical Computed Tomography angiography protocols, the samples were then removed from the scanner and a contrast agent (iodixanol 270 mg I/ml, Visipaque^TM^, GE Healthcare, USA) was added to the media (20-fold dilution). The tubes were inverted three times to ensure that the contrast agent was thoroughly mixed. Next, a contrast-enhanced CT (CECT) scan was made 5 min after administering the contrast agent to the tubes to quantify the contrast agent penetration into the clots. It was observed in preliminary analysis that the size of the region of interest (ROI) in relation to the size of the clot (diameter) affected the absolute CT density values calculated from CECT scans (Fig. S3). Therefore, for each clot, three circular non-overlapping regions of interest (ROI) were defined with a diameter half that of the sample diameter (as measured in Compressive Mechanical Characterisation). Within each ROI, the average Hounsfield Unit was recorded with ImageJ (1.53 k, National Institutes of Health), of which the overall average NCCT density for the 3 ROIs per clot was calculated. As a proxy for clinical perviousness measurements, the static CECT density increase after 5 min in the current study was quantified by subtracting the average CT density of the clot from the NCCT scan from the average CT density of the clot from the CECT scan (Fig. 1c). To account for any potential confounding effects of passive diffusion occurring in the CECT scans due to the effect of increased contraction and resultant smaller clot size, we also examined associations between clot size and CT density measurements (clot diameter in mm as determined in the Compressive Mechanical Characteristics section).

### Compressive mechanical characterisation

After imaging, the clots were rinsed in media and were then cut into 2 mm-thick sections. From each clot, at least one section was prepared for mechanical characterisation. In most cases, two clots were tested. A third section was used for histology and immunohistochemistry (Fig. 1d). To measure the cross-sectional area, the 2 mm-thick cross sections were photographed in a scaled image and the area and Feret’s diameters were measured using ImageJ. Each section was then placed on the stage of the compression tester in a 37 °C water bath filled with media. The clot was allowed to equilibrate for 5 min. Compression tests were performed using a custom-made compression tester, as previously described [3]. The sections were compressed to 80% strain, with a compression and retraction speed of 0.2 mm/s (10% strain/s) for 10 cycles [2]. The measured force was converted to nominal stress using the cross-sectional area of the samples. As a measure for the stiffness, the low and high-strain secant moduli were calculated by applying a linear fit to the stress-strain data between 0–10 and 75–80% strain, respectively. For viscoelasticity, the percent hysteresis loss was defined as the dissipated energy after the first loading–unloading cycle expressed as a percentage of the supplied energy through the loading cycle (area under the curve). The amount of plastic compressive strain (%) was also identified as the point where the force (N) returned to baseline on the unloading curve (Fig. 1e).

### Histology and immunohistochemistry

The RBC and content of the clots for comparison against the imaging and mechanical characteristics were determined using histology and immunohistochemistry. The sections were placed in 4% buffered formaldehyde for 48 h at 4 °C and embedded in paraffin wax. The sections were then cut into 5 μm slices. RBCs can be more easily detected from MSB images compared with traditional H&E images [4, 9], and therefore, Martius Scarlet Blue (MSB) stain was used to identify the clot RBC, fibrin/platelet and white blood cell content, as previously described [8–10]. Platelets can be accurately identified using immunohistochemistry techniques [3], specifically CD42b [9]. Therefore, CD42b (1:200) immunohistochemistry was used to measure the clot platelet content [3]. The samples were scanned at 40× magnification and 0.23 μm/pixel resolution (2.0 HT Nanozoomer, Hamamatsu, Japan) and components were quantified using Orbit Image Analysis software (Orbit; www.Orbit.bio) [10] (Fig. 1f). Where possible, the average of two slices was reported for each donor. RBC content (%) and platelet content (%) area were expressed as a percentage of the total clot cross-sectional area. RBC content (%) as determined from the MSB images and platelet content (%) as determined from the CD42b images are used for the regression analysis in this study. To compare the RBC and platelet contents to those published for AIS thrombi, normalized percentage clot composition (RBC, platelet and other (fibrin and white blood cells)) was determined by expressing the measured RBC and platelet areas as a percentage of the combined MSB clot area and CD42b platelet area.

### Statistical analysis

Shapiro–Wilk analysis was performed to assess the distribution of RBC and platelet clot content (%). Both clot contents were determined to be not normally distributed and were therefore summarised as median [25–75th]. Scatterplots were used to inspect the linearity of the relationship between histological components (RBCs and platelets) and the CT or mechanical characteristics. Independent associations of either RBC or platelet content (%) with imaging and mechanical characteristic were examined using simple (univariate) linear regression. The combined effect of RBC and platelet content on resulting clot imaging and mechanical characteristics were examined using least squares multiple linear regression. Regression results are summarised as unstandardised coefficient (β) [95% confidence interval]. All statistical analyses were conducted using GraphPad Prism 9.5.1. A *P* value < 0.05 was deemed statistically significant in the current study.

## Results

### Study population

A total of 6 healthy human donors were included in this study (3 males and 3 females, aged 23–49 years). Donor whole blood cell counts, and fibrinogen levels are presented in Table 1, which are all within the normal range for healthy human adults.

**Table 1:** Donor whole blood cell counts and fibrinogen levels

Donor #	Platelets(× 10^3^ platelets/μl)	Haematocrit (fraction)	White blood cells (× 10^3^ cells/μl)	Fibrinogen (g/l)
1	170	0.45	5.30	3.20
2	172	0.40	5.67	4.00
3	242	0.40	7.20	3.00
4	185	0.43	9.40	2.20
5	146	0.42	5.07	2.80
6	164	0.39	5.80	2.40

### Range of clot red blood cell and platelet contents

**Fig. 2:**
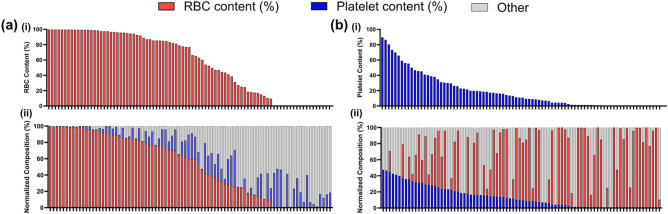
Percentage composition of the clot analogues. **a** Ordered from highest to lowest (i) red blood cell (RBC) content (%) as measured from the MSB images and (ii) normalized RBC composition (%) (red). **b** Order from highest to lowest (i) platelet content (%) as measured from the CD42b images and (ii) normalized platelet composition (%) (blue). Other clot components (fibrin and white blood cells) are indicated in grey

In total, 87 clots were produced for this study. The clot RBC content ranged from < 1% to 99.89% (Fig. 2a). The RBC median [Interquartile range] was 76.85 [15.06–96.13] %. The clot platelet content ranged from < 1 to 89.65% (10.83 [1.16–29.52] %) (Fig. 2b). The range of RBC and platelet content in the clot analogues produced in the current study periodically cover the range of RBC and platelet content observed for AIS thrombi retrieved with EVT (< 1% to 85% RBC and 3–88% platelet content [8]). Representative MSB and CD42b clots are shown in Fig. S4. Clot details are provided in Table S2.

### Clot composition and computed tomography characteristics

#### NCCT characteristics

**Fig. 3:**
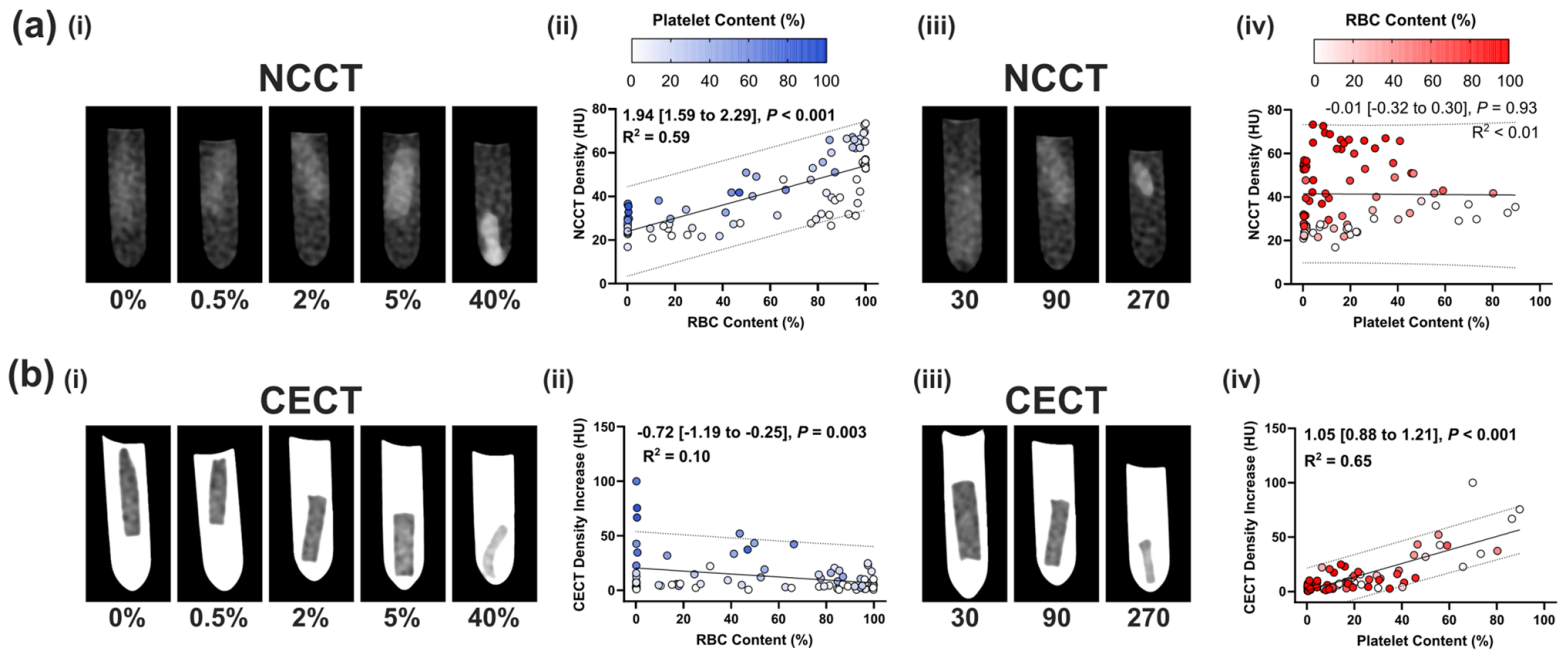
Clot computed tomography (CT) characteristics. **a** (i) Representative red blood cell (RBC) group CT images for a non-contrast (NCCT) and contrast-enhanced (CECT) scan (all 90 × 10^3^ platelets/μl). (ii) Scatterplots of RBC content versus NCCT density and CECT density increase. Points are coloured according to platelet content. **b** (i) Representative platelet concentration group CT images for a NCCT and CECT scan (all 2% RBC volume). (ii) Scatterplots of platelet content versus NCCT density and CECT density increase. Points are coloured according to RBC content. Simple linear regression 95% prediction lines are plotted. Simple linear regression results are presented on each scatterplot. Unstandardised regression coefficients (*β*) are presented with the 95% confidence interval in brackets. Significant regression values are represented in bold. *R*^2^ = goodness of fit

Individually, only the clot RBC content (%) was significantly associated with the NCCT clot density (*β*: 1.94 [Confidence Interval 1.59–2.29] (Fig. 3a(i)–(ii))). With every 1.94% increase in clot RBC content, the clot NCCT density increases by 1 HU. Platelet content was not associated with NCCT density in simple linear regression (Fig. 3a(iii)–(iv)). To assess the combined effect of RBC and platelet content on NCCT density, multiple linear regression was performed (Table 2). Considering the combined cellular effect, both RBC and platelet content were significantly positively associated with NCCT density (0.35 [0.30 to 0.40] and 0.22 [0.13 to 0.31], respectively, *P* < 0.001 for both) and the goodness of fit of the overall model was improved (*R*^2^ = 0.68 in multiple versus *R*^2^ = 0.59 for RBC in simple linear regression).

#### CECT density increase

For CECT density increase, in simple linear regression, both the RBC and platelet content were statistically associated with CECT density increase (RBC: − 0.72 [− 1.19 to − 0.25], *P* = 0.003 and Platelet: 1.05 [0.88 to 1.21], *P* < 0.001 (Fig. 3b)). However, both the *R*^2^ value and slope of the regression for RBC content associations were low (*R*^2^ = 0.10 and *β* = − 0.72) (Fig. 3b(ii)). In multiple linear regression, only the platelet content was significantly positively associated with CECT density increase (0.62 [0.51 to 0.73], *P* < 0.001, Table 2). RBC content was not correlated with CECT density increase in multiple regression analysis (Table 2). Smaller clots and higher platelet content were associated with CECT density increase in multiple linear regression analysis (− 10.23 [− 15.76 to − 4.70] and 0.49 [0.37 to 0.61], *P* < 0.001 for both) (Table S3).

**Table 2:** Multiple linear regression analysis of clot content (RBC and platelet) with CT imaging characteristics (density and density increase).

		R^2^	β0	β (95% CI)	*P* value
NCCT Density (HU)	RBC Content (%)	0.68	17.32	0.35 (0.30 to 0.40)	< 0.001
	Platelet Content (%)			0.22 (0.13 to 0.31)	< 0.001
CECT Density Increase HU)	RBC Content (%)	0.65	1.462	− 0.01 (− 0.07 to 0.05)	0.79
	Platelet Content (%)			0.62 (0.51 to 0.73)	< 0.001

Unstandardised regression coefficients (*β*) are presented with 95% confidence interval in brackets

*R*^2^ goodness of fit, *β*0 intercept

Significant values are represented in bold

### Clot composition and compressive mechanical characteristics

**Fig. 4:**
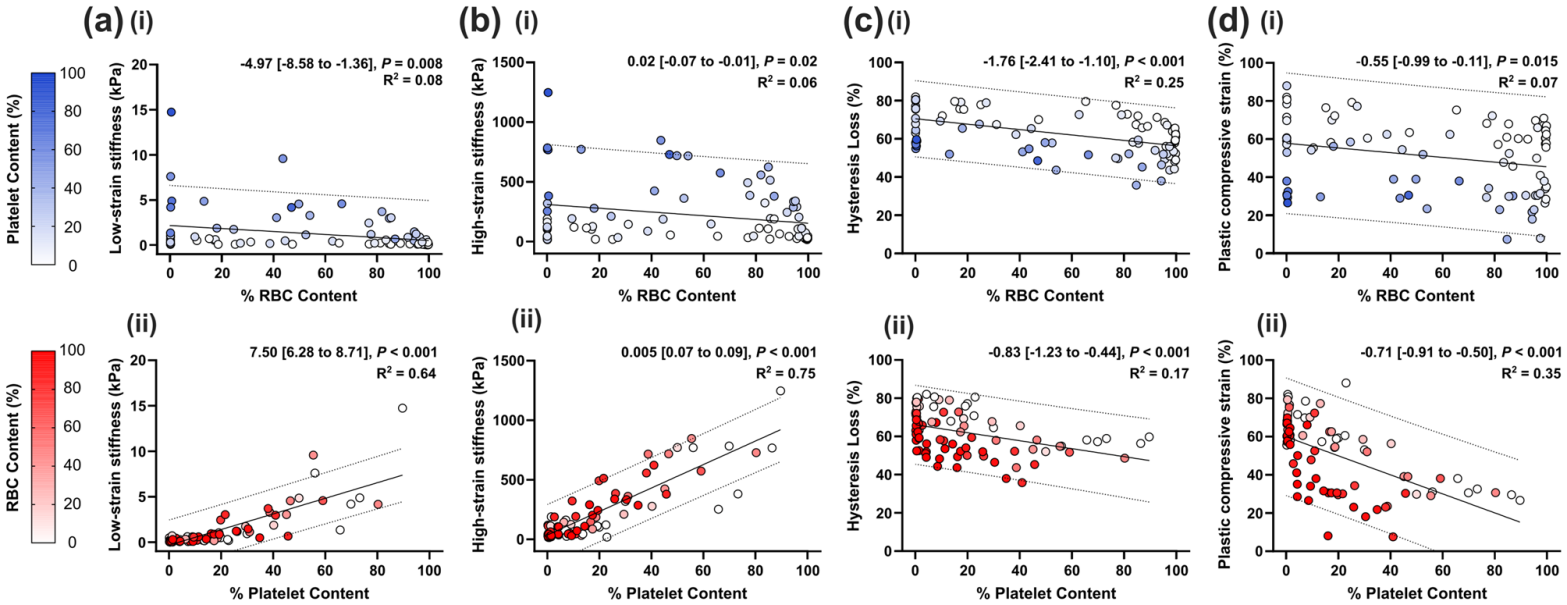
Clot mechanical characteristics. **a** low-strain stiffness (kPa), **b** high-strain stiffness (kPa), **c** hysteresis loss (%), and **d** plastic strain (%) for (i) red blood cell (RBC) content (%) and (ii) platelet content (%). Simple linear regression 95% prediction lines are plotted. Simple linear regression results are presented on each scatterplot. Unstandardised regression coefficients (*β*) are presented with the 95% confidence interval in brackets. Significant values are represented in bold. *R*^2^ = goodness of fit

Figure 4 presents the simple linear regression analyses of RBC and platelet content with the clot mechanical characteristics. Low- and high-strain clot stiffnesses appear to have similar results. Both RBC and platelet content were significantly associated with each of the mechanical characteristics (low- and high-strain stiffness (kPa), hysteresis loss (%) and plastic strain (%)) (Fig. 4). However, considering the regression coefficients (*β*) and goodness of fit (*R*^2^), individually, higher RBC content only appeared to reduce the observed hysteresis (− 1.76 [− 2.41 to − 1.10], *P* < 0.001 (Fig. 4c(i))). Higher platelet content increased the clot stiffness at both low- and high strains (Low: 7.50 [6.28 to 8.71], *P* < 0.001 and High: 0.005 [0.07 to 0.09], *P* < 0.001 (Fig. 4a, b(ii))) and additionally reduced the observed hysteresis (− 0.83 [− 1.23 to − 0.44], *P* < 0.001 (Fig. 4c(ii)) and compressive plastic strain (− 0.71 [− 0.91 to − 0.50], *P* < 0.001 (Fig. 4d(ii)). Considering the combined effect of RBC and platelet content in multiple linear regression (Table 3), only platelet content was significantly positively associated with clot stiffness, particularly at high strains (10.22 [8.92 to 11.53], *P* < 0.001). Higher platelet content resulted in stiffer clots. Measures of clot viscoelasticity (hysteresis loss (%)) and plastic deformation (plastic strain (%)) were determined by both RBC and platelet content in multiple linear regression (hysteresis loss: − 0.21 [− 0.25 to − 0.18] and − 0.35 [− 0.42 to − 0.28] plastic strain: − 0.26 [− 0.33 to − 0.19] and − 0.67 [− 0.79 to − 0.55], respectively, *P* < 0.001) (Table 3). Higher RBC and platelet content reduced the hysteresis loss and plastic strain observed during cyclic compression testing. Of note, one outlier data point was removed from the low-strain stiffness figure and analysis as the sample was inadvertently deformed prior to testing.

**Table 3:** Multiple linear regression analysis of clot content (RBC and platelet) with compressive mechanical characteristics (stiffness, hysteresis loss and plastic strain)

		*R*^2^	*β*0	*β* (95% CI)	*P* value
Low-strain stiffness (kPa)	RBC Content (%)	0.64	− 0.26	− 0.001 (− 0.001 to 0.01)	0.82
	Platelet Content (%)			0.09 (0.07 to 0.1)	< 0.001
High-strain stiffness (kPa)	RBC Content (%)	0.76	− 1.973	0.54 (− 0.19 to 1.27)	0.15
	Platelet Content (%)			10.22 (8.92 to 11.53)	< 0.001
Hysteresis loss (%)	RBC Content (%)	0.67	80.97	− 0.21 (− 0.25 to − 0.18)	< 0.001
	Platelet Content (%)			− 0.35 (− 0.42 to − 0.28)	< 0.001
Plastic strain (%)	RBC Content (%)	0.62	78.25	− 0.26 (− 0.33 to − 0.19)	< 0.001
	Platelet Content (%)			− 0.67 (− 0.79 to − 0.55)	< 0.001

Unstandardised regression coefficients (*β*) are presented with 95% confidence interval in brackets

*R*^2^ goodness of fit, *β*0 intercept

Significant values are represented in bold

## Discussion

In this study, we described a method for preparing human blood clot analogues with a range of RBC and platelet clot content representative of AIS thrombi retrieved from thrombectomy procedures. We then utilised these samples to analyse and decouple the individual and combined effects of both RBC and platelet content on clot (1) CT imaging and (2) compressive mechanical characteristics. NCCT density is primarily positively influenced by the clot RBC content; however, additional consideration of the platelet content improves the prediction of clot CT density. Higher platelet contents alone result in more pervious clots (higher CECT density increase). Additionally, higher platelet content is the major determinant of clot stiffness. Both RBC and platelet content contribute to reduced clot viscoelastic and plastic compressive behaviour. These in vitro observations should be confirmed in a clinical cohort.

As far as the authors are aware, this is the first study to examine the combined effect of RBC and platelet content on NCCT density. Notably, while RBC content is the dominant driver of NCCT density, additional analysis of the platelet content improved the association with higher CT density. Previous studies have demonstrated a positive association between thrombus RBC content and NCCT density [13, 20, 29, 33]. Importantly for the platelet content, in our simple linear regression analysis, we observed a non-significant, weak negative association between platelet content and NCCT density. This is in line with previously reported clinical CT associations between thrombus platelet content and NCCT density, where either no statistically significant associations or weak negative associations were reported [9, 20]. For the first time in the current study, we document the improved association observed between clot composition and NCCT density when considering the effect of both the RBC and platelet clot content. These results highlight the interdependence of thrombus composition (RBC and platelet) on associations with CT imaging characteristics.

Clinically, thrombus perviousness is assessed by determining the increase in density from NCCT to CT angiography and reflects the contrast agent penetration into the thrombus, and is a proxy for thrombus permeability [23]. Here, we observed that blood clot analogue platelet content was the primary cellular contributor to CECT density increase (in vitro perviousness unit). Previous in vivo perviousness findings have been conflicting, two studies reported that higher thrombus RBC content leads to decreased perviousness [2, 13, 21], while another reported that pervious thrombi had higher RBC content [1]. Another study looking at platelet content reported that higher platelet content leads to decreased perviousness [33]. We previously hypothesised that contracted clots would result in a lower contrast agent uptake [4]. Of note, we also investigated the effect of clot size on the CT imaging characteristics and found that clot size was significantly negatively associated with CECT density increase, while platelet content was positively associated with CECT density increase. Since platelets govern the clot contraction [14], this is likely due to the increased contraction we observed for clots with higher platelet content, leading to a reduction in the final clot size. Clinically, thrombus length has been associated with thrombus RBC content [13], suggesting that measures of clot size may be useful. Ultimately, perviousness is likely determined by a combination of the platelet-driven clot contraction [14, 19], RBC resistant to volume reduction [15, 25] and polyhedrocyte formation [27] that affects the overall clot contraction, size [25] and porosity. In line with this hypothesis, an ex vivo study demonstrated that porous thrombi are more pervious [12].

In the compressive mechanical analysis of the clots in the current study, an increased platelet content was the principal component driving clot stiffness, especially at high strains. Previous studies on the effect of thrombus/clot RBC content on mechanical properties do not consider interdependent of RBCs and platelet (particularly for blood clot analogues) [4]. Associations of platelets with clot mechanics have demonstrated that platelet-contracted clots are stiffer than non-contracted clots for all RBC volumes [15] and platelet concentration is linearly associated with clot elastic modulus [6]. For thrombi retrieved from thrombectomy procedures, a strong positive association was observed with thrombus stiffness, but only for high platelet content (> 70%) [3]. Our study highlights the interdependence of RBC and platelet clot content on resultant clot stiffness. We have demonstrated that platelet content is the dominant cellular component affecting clot stiffness, compared to RBC content. Instead, RBCs are known viscoelastic contributors to clot mechanics [11]. Here, we observed that together, both higher platelet and RBC content reduced the hysteresis loss and compressive plastic strain observed. In agreement with the findings here, previous rheometric analysis of contracting blood clots revealed that the addition of RBCs or platelets increased the ratio of viscous to elastic effects observed in the clots [26]. This is the first study to report that both RBC and platelet content contribute to compressive mechanical viscoelastic and plastic properties. In the absence of platelets, centrifugal forces have been used to compact clots [27], demonstrating the deformation potential of non-platelet-contracted clots.

The long-term goal of AIS thrombus research is to improve the procedural and thus functional outcomes for the patients. Clot composition has a direct effect on the mechanical thrombectomy device performance [34]. Thrombus characteristics acquired from CT neuroimaging performed upon patient presentation at the stroke treatment centre may be utilized to determine thrombus composition and consequent treatment efficacy. Currently in the clinic, NCCT density and thrombus length can explain 30% of the thrombus RBC content variability [13]. The results of the current study suggest that CT density is largely driven by the RBC content, but the explained variability could be further improved by additionally considering the thrombus platelet content. CECT perviousness appears to be related to the platelet content. Therefore, high perviousness, but low density values likely indicate a platelet-rich clot. However, due to the additional interdependence of platelet content on clot contraction and size, in combination with the observed strong association of clot size on clot perviousness in the current study, these results should be interpreted with caution and confirmed in a clinical AIS cohort. Based on the results of the pre-treatment imaging workflow, a thrombectomy device (aspiration catheter or stent retriever) and deployment approach can be selected given the predicted interaction with thrombus type to maximize procedural and functional outcomes. For instance, additional stent retriever embedding times improve strut integration in platelet/fibrin-rich clots [31]. In addition, AIS patients typically have impaired clot contraction [28]. As demonstrated by the current compressive plastic strain results, thrombi with a low platelet content and associated lack of contraction could be deformed via balloon angioplasty to relieve the vessel occlusion and restore blood flow.

The range of RBC and platelet content in the clot analogues produced in the current study periodically cover the range of RBC and platelet content observed in thrombi retrieved with EVT (< 1–90% RBCs [3, 8] and 3–88% platelets [8]). This blood clot model could be further improved by decreasing the clots with > 90% RBC content and < 3% platelet content. In blood clot analogue methodologies, especially those that utilize fractions of RBCs and PRP [15], RBC and platelet content will be linked as we observed here. However, RBC and platelet content also appear to be linked for AIS thrombi [7].Furthermore, the NCCT density range of the blood clot analogues in this study is comparable with clinical density values reported for AIS thrombi [13] (38.0 [27.2–54.5] HU versus 54.3 [46.4–59.6]).

The primary limitation with this study is that it is a static in vitro model; clots are prepared in a static manner and CT imaging is not conducted under flow. AIS thrombi are formed under different shear conditions that likely contribute to the heterogeneous composition of ex vivo AIS thrombi [3]. Future studies should consider the production of clots under flow using a Chandler Loop, for example, that may capture some of the in vivo thrombus features that are not considered in the current study. However, given the complexity of thrombosis in vivo, and the objective of the current study was to analyse the interdependence of RBC and platelets on imaging and mechanical properties, we produced clots in a static manner to (1) control for as many variables as possible and to (2) simplify the analysis of the imaging and mechanical results. There are also some important differences between in vivo CTA perviousness and in vitro CECT density increase as described in the current study. In vitro, the clots are statically submerged contrast agent and are not exposed to in vivo blood pressure and flow. This may affect the diffusion and/or uptake of contrast agent into the clots. Therefore, we refrained from using the term ‘perviousness’ and instead report CECT density increase. In general, the results of this static in vitro study should be confirmed in a clinical AIS population. There are two other minor limitations with this research that should be considered. First, we did not control for fibrin(ogen) levels in our cohort. However, soluble proteins such as fibrinogen are not expected pelleted out during centrifugation. Therefore, our PRP and PDP plasma samples should have the same fibrinogen concentration within donors. Second, we previously demonstrated that human blood clot analogues behave differently under compressive versus tensile loading [5]. However, a limited citrated whole blood volume is collected from each donor, which is insufficient to produce the wide range of clots with differing RBC and platelet content in both compressive and tensile formats. Future work is warranted to investigate the interdependence of RBC and platelet content on tensile blood clot behaviours. Third, the donors in this study were healthy human adults who likely have normal platelet function. However, AIS patients exhibit impaired clot contraction [28]. Therefore, platelet functional assessment such as a platelet-contraction cytometer [19] could be combined with perviousness estimations of platelet content to provide a more holistic account of thrombus contraction.

Clot RBC and platelet content are linked, and this relationship should be considered in the analysis of AIS CT imaging and mechanical characteristics. CT density is predominantly determined by the clot RBC content, but explanation of variability could be improved by considering the additional effect of clot platelet content. Higher platelet content determines the clot perviousness as well as compressive stiffness. However, review of additional in vitro factors (e.g. clot contraction and size) should be considered when examining platelet effects. Both RBC and platelet content contribute to reduced compressive clot viscoelasticity and plastic strain. The results of the current study should be confirmed in a clinical AIS cohort.

### Supplementary Information

Below is the link to the electronic supplementary material.

Supplementary file1 (DOCX 3336 kb)

## Abbreviations

AIS: Acute ischaemic stroke
CECT: Contrast-enhanced computed tomography
EVT: Endovascular thrombectomy
HU: Hounsfield unit
MSB: Martius scarlet blue
NCCT: Non-contrast computed tomography
PDP: Platelet-depleted plasma
PRP: Platelet-rich plasma
