# Supplementary material for: Contribution of Red Blood Cells and Platelets to Blood Clot Computed Tomography Imaging and Compressive Mechanical Characteristics
Source: Ann Biomed Eng. 2024 Apr 25;52(8):2151–61. doi: 10.1007/s10439-024-03515-y (PMC11247058; doi:10.1007/s10439-024-03515-y)
Supplement: Supplementary file 1 — Supplementary file1 (DOCX 3336 kb) [file 10439_2024_3515_MOESM1_ESM.docx]

**Contribution of red blood cells and platelets to blood clot computed tomography imaging and compressive mechanical characteristics**

**SUPPLEMENTARY FILE**

# Authors and Institutions

Rachel M.E. Cahalane^1^, Janneke M.H. Cruts^1^, Heleen M.M. van Beusekom^2^, Moniek P.M. de Maat^3^, Marcel Dijkshoorn^4^, Aad van der Lugt^4^, Frank J.H. Gijsen^1,5^

^1^ Department of Biomedical Engineering, Thoraxcenter, Erasmus MC, Rotterdam, the Netherlands.

^2^ Experimental Cardiology, Erasmus Medical Center, Rotterdam, The Netherlands.

^3^ Department of Hematology, Erasmus MC, University Medical Center Rotterdam, Rotterdam, The Netherlands

^4^ Department of Radiology and Nuclear Medicine, Erasmus MC, University Medical Center Rotterdam, Rotterdam, the Netherlands

^5^ Department of Biomechanical Engineering, Delft University of Technology, Delft, the Netherlands.

# Corresponding author details

F. Gijsen, PhD, Department of Bioengineering, Erasmus MC, University Medical Center, PO Box 2040 3000 CA Rotterdam, the Netherlands

e-mail: [f.gijsen@erasmusmc.nl](mailto:f.gijsen@erasmusmc.nl)

# SUPPLEMENTAL MATERIALS AND METHODS

## Blood Cell Count and Hemostasis

Hemoglobin, RBC, white blood cells, and platelet levels were measured in the whole blood sample using a Coulter Counter. The fibrinogen level was quantified in PDP using a Clauss Assay after undergoing one freeze-thaw cycle, diluted 2-fold and calculated using Clot Wave Analysis.

**RBC Volume and Platelet Concentration versus the Degree of Gravimetric Clot Contraction**

Blood was drawn by venepuncture from three healthy human volunteers (2 males, 1 female, age range 24 – 54 years). To determine the effect of platelet concentration on the degree of clot contraction the PRP and PDP were reconstructed in 12 different concentration 0, 10, 20, 30, 40, 50, 60, 70, 80, 100, 200, 400 (300 for one donor) x 103 platelets/μl. All clots were prepared with 5% RBC volume. The reconstructed blood was immediately transferred into 3mL syringes and placed vertically in a 37 ̊C water bath to fully contract for at least 2 hrs. To assess the degree of gravimetric clot contraction, the weight of the expelled serum was expressed as a percentage of the weight of the total reconstructed blood, as previously described.^1^

## Computed Tomography Region of Interest Size

To assess the effect of Region of Interest size on the CT density results, we analysed all clots from one representative donor on the NCCT scans and the CECT scan 5 mins after administering the contrast agent. For each clot, three non-overlapping regions of interest with diameters equal to 1, ½ , ¾ and ¼ of the sample diameter as measured in Compressive Mechanical Characterisation were utilised.

## Expelled Serum Platelet Concentration

Blood was drawn by venepuncture from two healthy human volunteers (male, 29 and 50 years) into 0.109M buffered sodium citrate tubes (BD Vacutainer®). An aliquot of the reconstructed blood for each sample was set aside for cell counting. The reconstructed blood aliquots were kept at room temperature for 2 hrs. After 2 hrs, the clots were removed from the syringes and both the reconstructed blood aliquot and expelled serum were taken for cell counting.

# FIGURES


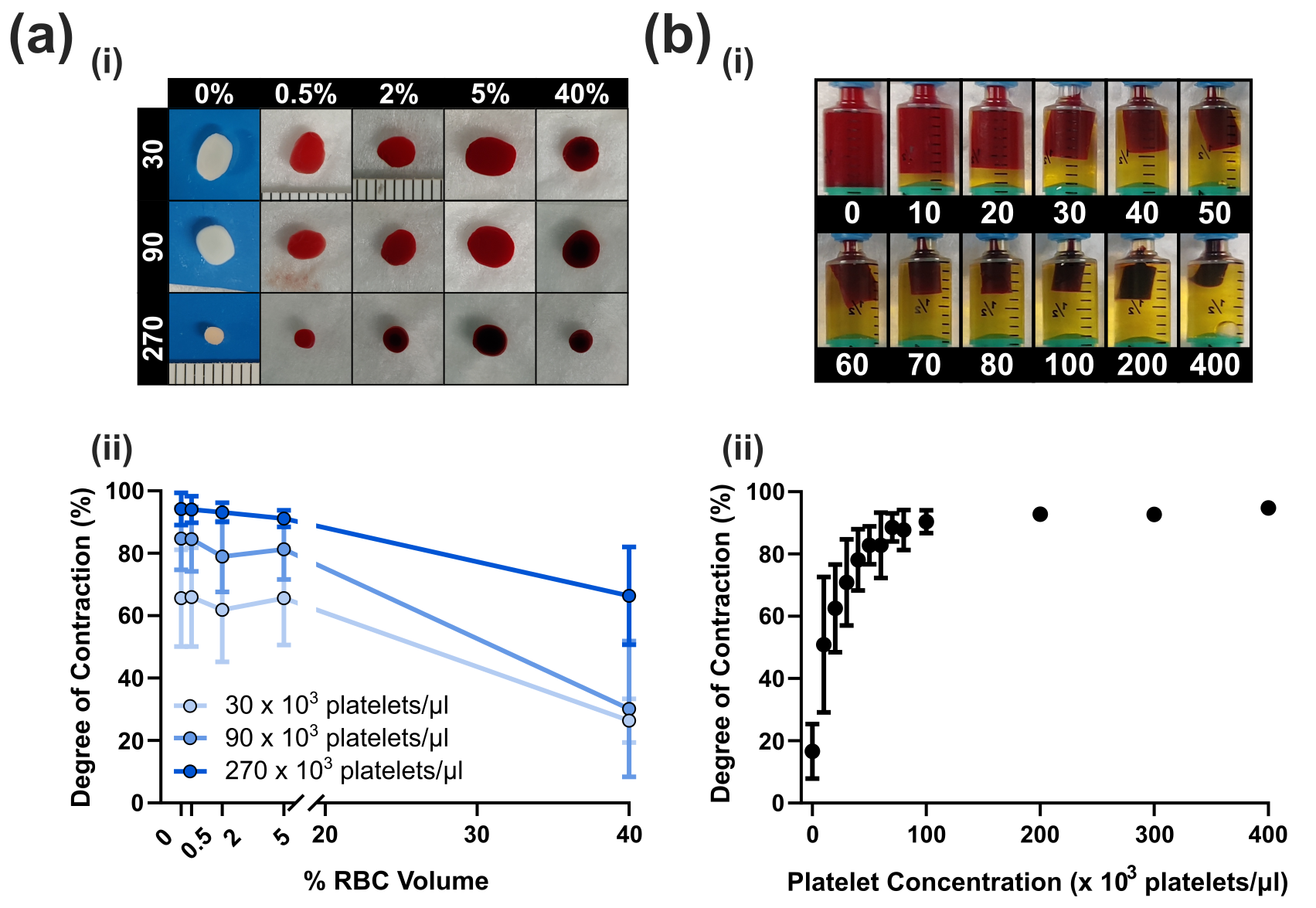


**Figure S1: Red blood cell and platelet concentrations are negatively linearly and positively non-linearly associated with the degree of clot contraction.** (a) (i) Representative cross sectional area photos for each clot type. Boxes indicate 1 x 1 cm dimensions. (ii) Red blood cell volume group and the final degree of clot contraction per platelet concentration (30, 90 and 270 x 10^3^ platelets/μl). (b) (i) Representative images of contracted clots in syringes. (ii) Platelet concentration and the final degree of clot contraction for 5% RBC volume clots.


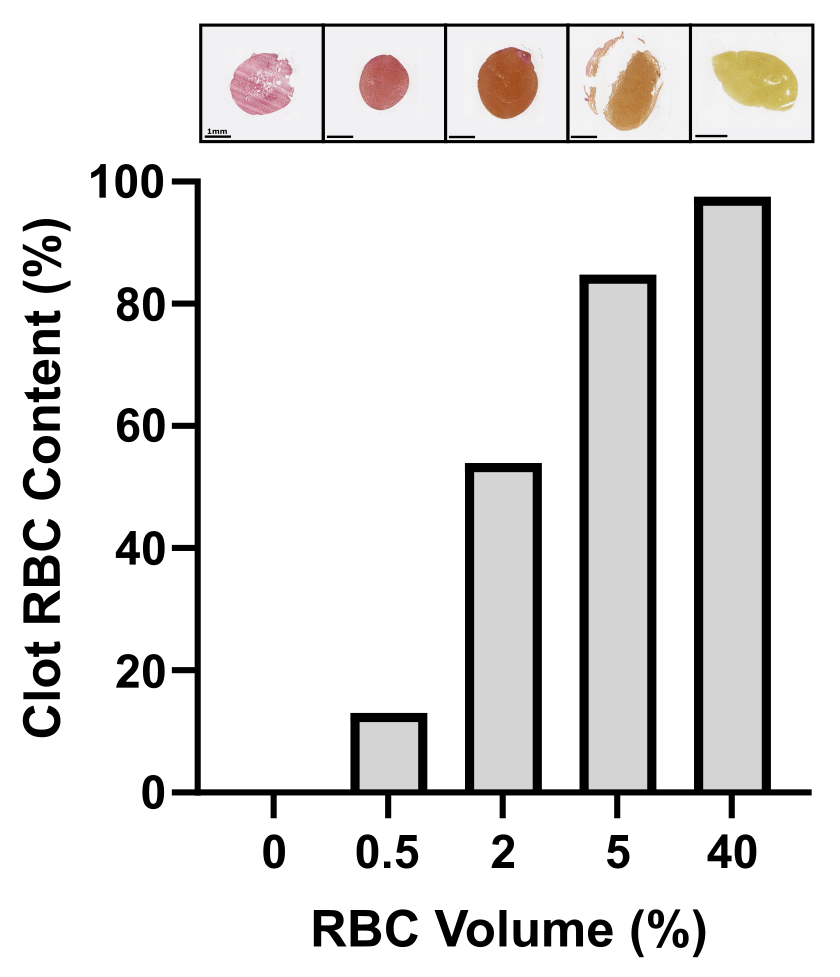


**Figure S2: Red blood cell content is not proportional to concentration.** Preliminary data on the reconstructed blood red blood cell (RBC) volumes used in the current study versus resulting clot RBC content (n=1). Values for samples with a platelet concentration of 270 x 10^3^ platelets/μl are presented. Representative Martius Scarlet Blue staining is displayed. All scale bars indicate 1 mm.


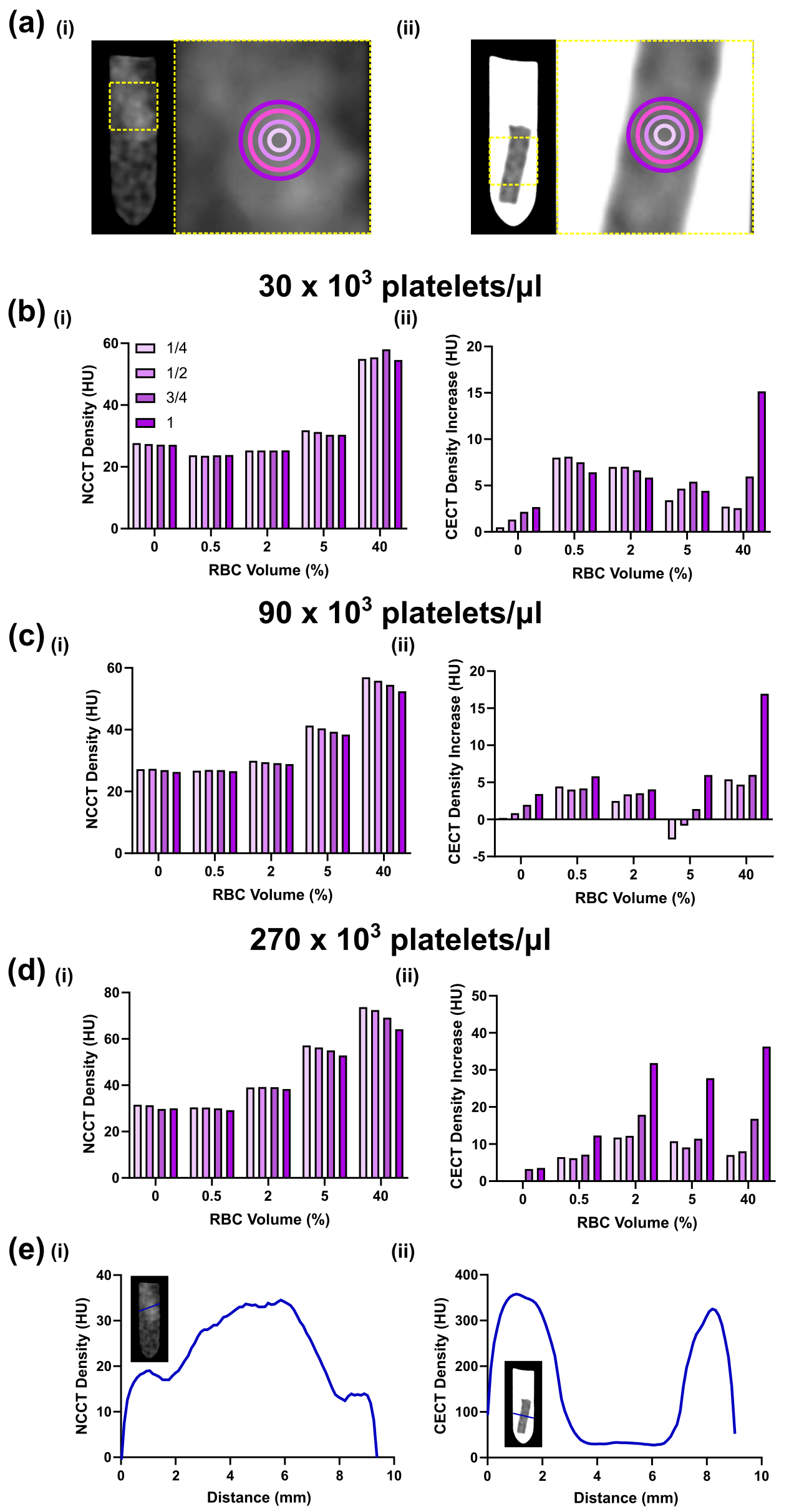


**Figure S3:** **Contrast enhanced computed tomography increase in density measurements are affected by the size of the region of interest.** (a) 2% RBC volume, 90 x 10^3^ platelets/μl sample (i) NCCT and (ii) CECT after 5 mins. The size of ROIs used were ¼, ½, ¾ and 1 times the Feret’s diameter as calculated in Section 2.4. (b) 30, (c) 90 and (d) 270 x 10^3^ platelets/μl groups with (i) NCCT density and (ii) CECT absolute density calculations. (e) Changes in absolute density across the tube and sample for a 2% RBC volume, 90 x 10^3^ platelets/μl sample in (i) NCCT scan and (ii) CECT scan after 5 mins.


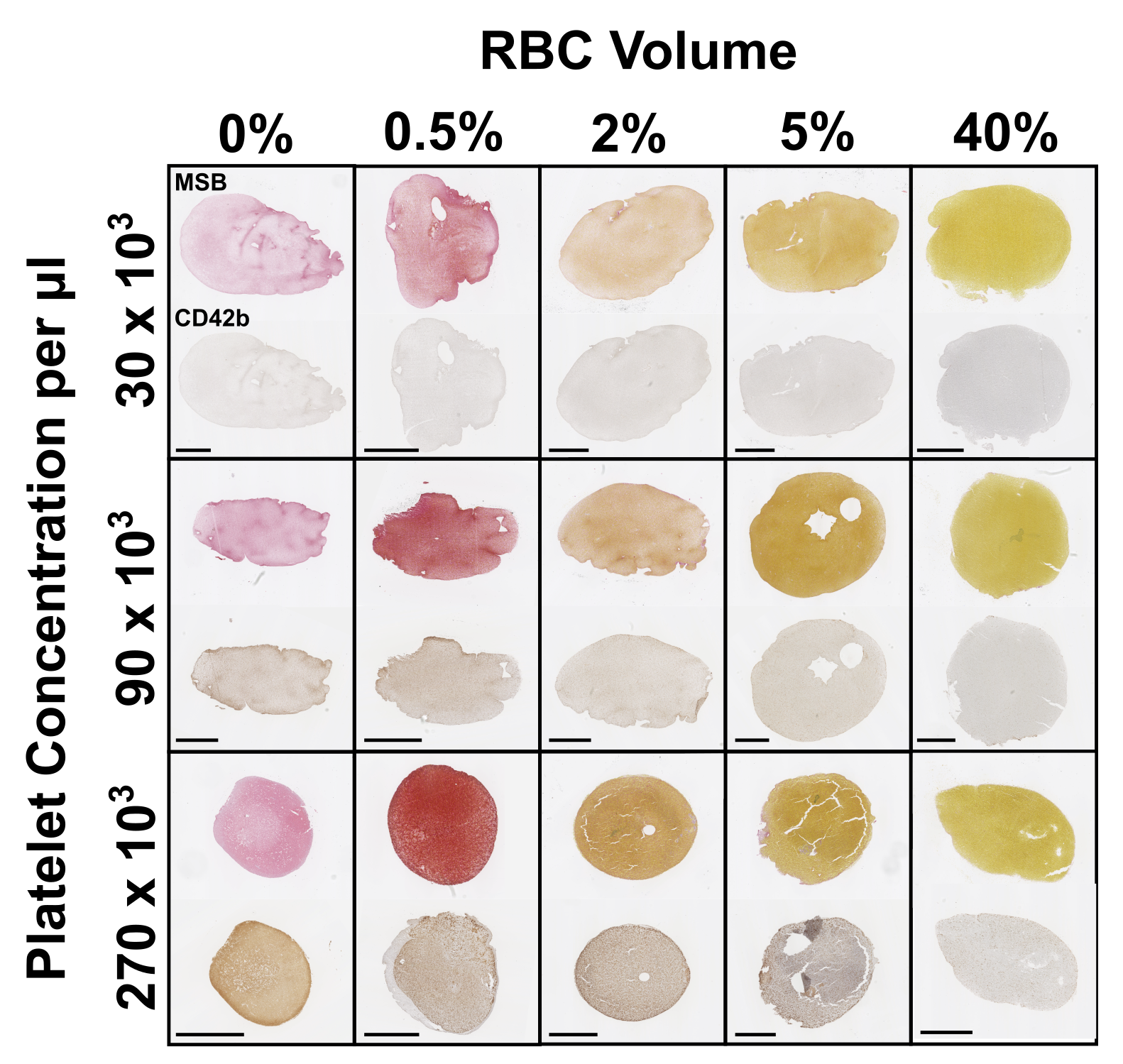


**Figure S4:** **Representative histology sections for the 15 clot types in the current study.** Martius Scarlet blue (MSB) (top) and CD42b (bottom). Scale bars represent 1mm.

# TABLES

**Table S1:** Example reconstructed blood volume calculations. A platelet-rich plasma (PRP) concentration of 758 x 10^3^ platelets/μl is used in the current example.

| Platelet Concentration (x 10^3^ platelets/μl) | RBC Volume  (%) | Total Plasma Required  (μl) | PRP volume (758 x 10^3^ platelets/μl concentration)  (μl) | PDP (μl) | RBCs (μl) | CaCl_2_ (850mM stock) (μl) | Thrombin (100U/mL stock) (μl) | Final Volume  (μl) | Syringe Size |
| --- | --- | --- | --- | --- | --- | --- | --- | --- | --- |
| 30 | 0 | 1455 | 60 | 1395 | 0 | 30 | 15 | 1500 | 3mL |
|  | 0.5 | 1447.5 | 60 | 1387 | 7.5 | 30 | 15 | 1500 | 3mL |
|  | 2 | 1425 | 60 | 1365 | 30 | 30 | 15 | 1500 | 3mL |
|  | 5 | 1380 | 60 | 1320 | 75 | 30 | 15 | 1500 | 3mL |
|  | 40 | 285 | 20 | 265 | 200 | 10 | 5 | 500 | 1mL |
| 90 | 0 | 1940 | 241 | 1699 | 0 | 40 | 20 | 2000 | 3mL |
|  | 0.5 | 1447.5 | 180 | 1267 | 7.5 | 30 | 15 | 1500 | 3mL |
|  | 2 | 1425 | 180 | 1245 | 30 | 30 | 15 | 1500 | 3mL |
|  | 5 | 1380 | 180 | 1200 | 75 | 30 | 15 | 1500 | 3mL |
|  | 40 | 285 | 60 | 225 | 200 | 10 | 5 | 500 | 1mL |
| 270 | 0 | 1940 | 722 | 1218 | 0 | 40 | 20 | 2000 | 3mL |
|  | 0.5 | 1930 | 722 | 1208 | 10 | 40 | 20 | 2000 | 3mL |
|  | 2 | 1425 | 541 | 884 | 30 | 30 | 15 | 1500 | 3mL |
|  | 5 | 1380 | 541 | 839 | 75 | 30 | 15 | 1500 | 3mL |
|  | 40 | 285 | 180 | 105 | 200 | 10 | 5 | 500 | 1mL |

**Table S2:** Samples prepared and analysed in this study. For one of the donors, not enough plasma could be isolated to prepare all the clot types. Therefore no 2% RBC volume samples were prepared for this donor (5).

| RBC Volume (%) | Platelet Concentration (x10^3^ platelets/μl) | Number of Donors |
| --- | --- | --- |
| 0 | 30 | 6 |
| 0 | 90 | 6 |
| 0 | 270 | 6 |
| 0.5 | 30 | 6 |
| 0.5 | 90 | 6 |
| 0.5 | 270 | 6 |
| 2 | 30 | 5 |
| 2 | 90 | 5 |
| 2 | 270 | 5 |
| 5 | 30 | 6 |
| 5 | 90 | 6 |
| 5 | 270 | 6 |
| 40 | 30 | 6 |
| 40 | 90 | 6 |
| 40 | 270 | 6 |

**Table S3:** Multiple linear regression analysis of clot content (RBC and platelet) and clot size (clot cross-sectional diameter in mm) with CT imaging characteristics (NCCT density and CECT density increase). Unstandardised regression coefficients (β) are presented with 95% confidence interval in brackets. Significant values are represented in bold. R^2^ = goodness of fit. β0 = intercept.

|  |  | **R^2^** | **β0** | **β (95% Confidence Interval)** | ***P*-value** |
| --- | --- | --- | --- | --- | --- |
| **NCCT Density (HU)** | **RBC Content (%)** | **0.69** | **31.36** | **0.38 (0.32 to 0.44)** | **< 0.001** |
|  | **Platelet Content (%)** |  |  | **0.16 (0.05 to 0.27)** | **0.004** |
|  | **Clot size (mm)** |  |  | -4.73 (-9.80 to 0.34) | 0.067 |
| **CECT Density Increase (HU)** | **RBC Content (%)** | **0.70** | **31.78** | 0.06 (-0.01 to 0.13) | 0.069 |
|  | **Platelet Content (%)** |  |  | **0.49 (0.37 to 0.61)** | **< 0.001** |
|  | **Clot size (mm)** |  |  | **-10.23 (-15.76 to -4.70)** | **< 0.001** |

# SUPPLEMENTAL REFERENCES

1. Johnson, S., J. Chueh, M. J. Gounis, R. McCarthy, J. P. McGarry, P. E. McHugh, and M. Gilvarry. Mechanical behavior of in vitro blood clots and the implications for acute ischemic stroke treatment. *J NeuroIntervent Surg* 12:853–857, 2020.
